# Microporous Materials Based on Norbornadiene-Based Cross-Linked Polymers

**DOI:** 10.3390/polym10121382

**Published:** 2018-12-13

**Authors:** Dmitry A. Alentiev, Dariya M. Dzhaparidze, Natalia N. Gavrilova, Victor P. Shantarovich, Elena V. Kiseleva, Maxim A. Topchiy, Andrey F. Asachenko, Pavel S. Gribanov, Mikhail S. Nechaev, Sergey A. Legkov, Galina N. Bondarenko, Maxim V. Bermeshev

**Affiliations:** 1 A.V. Topchiev Institute of Petrochemical Synthesis, Russian Academy of Sciences, 29 Leninsky prospekt, 119991 Moscow, Russia; d.alentiev@ips.ac.ru (D.A.A.); daria1000@mail.ru (D.M.D.); maxtopchiy@ya.ru (M.A.T.); asandrey@yandex.ru (A.F.A.); gribanovps@mail.ru (P.S.G.); mikhail.s.nechaev@hotmail.com (M.S.N.); legkov@ips.ac.ru (S.A.L.); bond@ips.ac.ru (G.N.B.); 2 Department of Chemistry and Technology of Polymer Materials and Nanocomposites, A.N. Kosygin Russian State University, 33-1 Sadovnicheskaya st., 117997 Moscow, Russia; 3 Department of Natural Sciences, D.I. Mendeleev University of Chemical Technology of Russia, 9 Miusskaya sq., 125047 Moscow, Russia; gavrilovann@yahoo.com; 4 N.N. Semenov Institute of Chemical Physics, Russian Academy of Sciences, 4 Kosygina st., 119991 Moscow, Russia; shant@center.chph.ras.ru (V.P.S.); kiseleva@polymer.chph.ras.ru (E.V.K.); 5 Chemistry Department, M.V. Lomonosov Moscow State University, 1-3 Leninskie gory, 119991 Moscow, Russia

**Keywords:** norbornadiene, oligomers of norbornadiene, addition polymerization, microporous polymers, porous organic materials

## Abstract

New microporous homopolymers were readily prepared from norbornadiene-2,5, its dimer and trimer by addition (vinyl) polymerization of the corresponding monomers with 60–98% yields. As a catalyst Pd-*N*-heterocyclic carbene complex or Ni(II) 2-ethylhexanoate activated with Na^+^[B(3,5-(CF_3_)_2_C_6_H_3_)_4_]^−^ or methylaluminoxane was used. The synthesized polynorbornenes are cross-linked and insoluble. They are glassy and amorphous polymers. Depending on the nature of the catalyst applied, BET surface areas were in the range of 420–970 m^2^/g. The polymers with the highest surface area were obtained in the presence of Pd-catalysts from the trimer of norbornadiene-2,5. The total pore volume of the polymers varies from 0.39 to 0.79 cm^3^/g, while the true volume of micropores was 0.14–0.16 cm^3^/g according to t-plot. These polymers gave CO_2_ uptake from 1.2 to 1.9 mmol/g at 273 K and 1 atm. The porous structure of new polymers was also studied by means of wide-angle X-ray diffraction and positron annihilation lifetime spectroscopy.

## 1. Introduction

**Figure 1:**
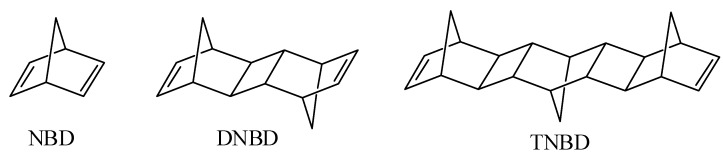
Bifunctional norbornene-type monomers: norbornadiene-2,5 (**NBD**), norbornadiene-2,5 dimer (**DNBD**) and norborbadiene-2,5 trimer (**TNBD**).

Microporous materials are attractive materials for gas storage, heterogeneous catalysis, membrane gas separation and so on. Crystalline zeolites and amorphous activated carbons are the most known examples of such materials. However, their opportunities (e.g., for hydrogen and hydrocarbon storage) are limited, therefore, the design of new microporous materials is a problem. In recent decades, a wide range of such materials with different properties have been developed [1,2]. For example, metal-organic frameworks (MOFs) and covalent-organic frameworks (COFs) possess crystalline order and narrow pore size distribution, hence, they are organic analogues of zeolites [3,4]. Another example is amorphous porous polymeric materials, for instance rigid-chain linear polymers such as polyacetylenes [5], polymers of intrinsic microporosity (PIMs) [6], TR-polymers [7], ferrocene-based polymers [8], polynorbornenes [9,10], and network polymers, such as cross-linked PIMs [11], aromatic conjugated microporous polymers [12] and polyolefins [13]. These polymers possess high surface areas and wide synthetic opportunities for modifications, and are therefore of great interest as membranes or gas storage materials. Polynorbornenes are of particular interest because of several advantages. Firstly, norbornenes could be easily modified which makes possible the introduction of a wide range of substituents within polymers. Secondly, norbornenes are capable of being polymerized by different mechanisms (metathesis [14], addition [15,16] or isomerization [17,18] polymerization), which results in polymers with a different main chain structure and chain rigidity, and hence various properties. This makes polynorbornenes attractive to design new polymers with desired properties [19,20]. Earlier we have shown that Me_3_Si-substituted addition polynorbornenes, being rigid-chain linear polymers, possess BET (obtained by Brunauer-Emmett-Teller method) surface area up to 780 m^2^/g, and are promising materials for membrane gas separation of hydrocarbons or CO_2_/N_2_ because of their extremely high gas permeability and/or high separation selectivities [21,22,23]. Herein we report the synthesis and properties of novel network amorphous porous polymers based on bifunctional norbornene monomers, namely norborbornadiene-2,5 and its oligomers (Figure 1). These monomers are more synthetically available than earlier studied silicon-substituted norbornenes, and the presence of two double bonds in them allows for creating various branched and network porous polymer structures depending on polymerization conditions and the size of monomer (the distance between double bonds in a monomer influences the length of cross-linkages). So, their polymerization could provide a simple approach to new microporous organic materials for various applications, especially gas storage and as fillers for modifications of gas separation composite membranes.

## 2. Experimental

### 2.1. Materials and Methods

All solvents, norbornadiene-2,5, sodium tetrakis[3,5-bis(trifluorumethyl)phenyl]borate (NaBARF), tricyclohexylphosphine (PCy_3_), nickel ethylhexanoate, methylaluminoxane, the 1st generation Grubbs catalyst, *p*-toluenesulfonyl hydrazide and other chemicals were purchased from Aldrich. 1,2-Dichloroethane was dried over P_2_O_5_, toluene was dried over sodium, *o*-xylene was dried over CaH_2_. Pd-*N*-heterocyclic carbene complex SIPrPd(cinn)Cl was synthesized according to previously published procedures [24,25]. Norbornadiene-2,5 dimer and trimer were synthesized by oligomerization of norbornadiene-2,5 in the presence of Ni(COD)_2_ [26] and separated by recrystallyzation from ethanol.

CP/MAS ^13^C NMR spectra were recorded at 125 MHz and at 25 °C on Varian Unity Inova AS500 spectrometer (Agilent Technologies, Santa Clara, CA, USA) equipped with a solid-state high-resolution apparatus. X-ray diffraction (WAXD) data were obtained using a two-coordinate AXS detector (Bruker, Karlsruhe, Germany) and Cu Kα emission (wavelength of 0.154 nm). IR spectra were recorded using IFS 66v/s Bruker spectrometer on KBr plates.

Characteristics of the porous structure of polymers were determined by nitrogen (77 K) and carbon dioxide (273 K) adsorption using a Gemini VIIt analyzer (Micromeritics Instrument Corporation, Norcross, GA, USA). All the samples were degassed at 100 °C at 25–50 mTorr for 10 h before measurements. Surface area was calculated by BET (N_2_) and Langmuir (CO_2_) equations. The calculations of *S*_BET_ were performed in the range of relative pressure (*p*/*p*_0_) of 0.08–0.22 in the case of polyDNBD synthesized over Ni-catalyst, and 0.03–0.15 in all other cases. Mesopores size distribution were calculated using the BJH (Barrett-Joyner-Halenda) method. The Harkins-Jura equation was used for adsorption film thickness calculation. Micropore volume and surface were determined using the Dubinin equation. Micropore size distribution was estimated using the Horvath-Kawazoe method. The T-plot method was used for external surface area and micropore volume value calculation.

The concentration of double bonds in polymers was measured by ozonation method on the compact original device “Double Bonds Analyzer” (DBA) which has ozone sensitivity on 10^−8^ mol/L, precision up to 0.1 % and short analysis time from 1 to 2 min [27].

The amount of double bonds in polyDNBD was also estimated by IR spectroscopy. The intensities of peaks at 3066 or 1562 cm^−1^ relative to the intensity of peak at 1451 cm^−1^ for polymers were compared with corresponding values for the monomer (see Figure S1).

### 2.2. Positron Annihilation Lifetime Spectroscopy (PALS)

The positron annihilation lifetime decay curves were measured at room temperature using an EG@GOrtec “fast-fast” lifetime spectrometer. A nickel-foil-supported [^44^Ti] radioactive positron source was used. Two stacks of film samples, each with a total thickness of about 1 mm, were placed on either side of the source. All the measurements were performed in inert (nitrogen) atmosphere. The time resolution was 300 ps (full width at the halfmaximum (fwhm) of the prompt coincidence curve). The contribution from annihilation in the source material, a background, and instrumental resolution were taken into account in the PATFIT program for treating the experimental lifetime data. The resulting data were determined as an average value from the several spectra collected for the same sample, having an integral number of counts of at least 10^6^ in each spectrum.

PALS is based on the measurements of positron lifetime spectra in polymers—lifetimes *τ_i_* (ns) and corresponding intensities *I_i_* (%). Longer lifetimes *τ*_3_ and *τ*_4_ can be related to the mean size of free volume elements (FVE) in polymers according to the Tao-Eldrup formula: [28,29]

$$\tau_{i}={\left\{\lambda_{0}^{T}+2\left[1-\frac{R_{i}}{R_{i}+\Delta R}+\frac{1}{2\pi }\sin\left(\frac{2\pi R_{i}}{R_{i}+\Delta R}\right)\right]\right\}}^{-1},$$

where *τ_i_* = *τ*_3_ or *τ*_4_ are o-Ps lifetimes and *R_i_* = *R*_3_ or *R*_4_ are the radii of free volume elements expressed in nanoseconds and angstroms respectively; $\lambda_{0}^{T}$—stands for the intrinsic *ortho*-Ps annihilation rate (0.7·10^9^ s^−1^); Δ*R* = 1.66 Å is the fitted empirical parameter.

### 2.3. General Procedure for Addition Homopolymerization Using Pd-Based Catalyst

The procedure is described for the polymerization of norbornadiene-2,5 dimer (DNBD) at the monomer/SIPrPd(cinn)Cl/NaBARF/PCy_3_ ratio of 1000/1/5/2 and the monomer concentration of 0.38 M using absolute 1,2-dichloroethane as a solvent. The polymerization of norbornadiene-2,5 (NBD) and norborbadiene-2,5 trimer (TNBD) was conducted by the similar procedure, excluding dissolution of TNBD and the purification stage for NBD (see below).

The catalytic system solution was prepared immediately prior to polymerization. For this absolute 1,2-dichloroethane solution of SIPrPd(cinn)Cl (6.77 mL, 6.77·10^−3^ mmol, 1.00·10^−3^ M), absolute 1,2-dichloroethane solution of NaBARF (5.55 mL, 3.39·10^−2^ mmol, 6.10·10^−2^ M), and absolute 1,2-dichloroethane solution of PCy_3_ (4.44 mL, 1.35·10^−2^ mmol, 3.05·10^−3^ M) were mixed in a Schlenk flask and stirred for 5 min. DNBD (0.50 g, 2.71 mmol) was dissolved in absolute 1,2-dichloroethane (0.5 mL) in a vial. The polymerization was initiated by the addition of catalytic system solution (6.7 mL) to the monomer solution while stirring. Then the reaction mixture was allowed to stay for 24 h. The immobile white mass formed was soxhleted with chloroform for 3 h and dried under vacuum at 60–80 °C up to a constant weight. Yield: 0.50 g (100 %). ^13^C NMR (CP/MAS, δ, ppm): 139.3–132.4 (C=C), 62.0–37.0, 36.5–26.1.

In the case of NBD, at the purification stage the viscous solution formed was evaporated in a vacuum, the brown solid formed was soxhleted with chloroform for 3 h and dried in a vacuum at 60–80 °C up to a constant weight. Yield: 0.057 g (53 %).

TNBD (0.10 g, 0.36 mmol) was dissolved in absolute toluene (0.4 mL) and diluted by 1,2-dichloroethane (0.5 mL). Then the polymerization was carried out similar to that of DNBD. Yield: 0.096 g (96 %). ^13^C NMR (CP/MAS, δ, ppm): 139.1–132.3 (C=C), 63.9–37.6, 36.1–26.2.

### 2.4. Addition Homopolymerization of DNBD Using Ni-Based Catalyst

DNBD (0.20 g, 1.09 mmol) was dissolved in absolute 1,2-dichloroethane (0.5 mL). The solution was introduced to a vial preliminary filled by argon. The solution of nickel ethylhexanoate in absolute toluene (0.08 mL, 3.5·10^−4^ mmol, 4.4·10^−3^ M), NaBARF absolute toluene solution (0.13 mL, 1.04·10^−3^ mmol, 8.0·10^−3^ M) and methylaluminoxane absolute toluene solution (0.06 mL, 7.8·10^−2^ mmol, 1.3 M) were subsequently introduced into the vial while stirring. Then the reaction mixture was allowed to stay for 24 h. The immobile colorless mass formed was soxhleted with chloroform for 3 h and dried in vacuum at 60–80 °C up to a constant weight. Yield: 0.20 g (100 %). ^13^C NMR (CP/MAS, δ, ppm): 139.2–131.9 (C=C), 56.3–37.0, 36.1–27.0.

### 2.5. Metathesis (ROMP) Homopolymerization of DNBD

DNBD (0.10 g, 5.43 mmol) was dissolved in absolute 1,2-dichloroethane (2 mL) in a vial. The polymerization was initiated by the addition of the 1st generation Grubbs catalyst solution in 1,2-dichloroethane (0.10 mL, 1.8·10^−3^ mmol, 1.8·10^−2^ M) while stirring. Then the reaction mixture was allowed to stay for 24 h. The white precipitate formed was isolated, rinsed by hexane (3 × 20 mL), and dried in a vacuum at 40–60 °C up to a constant weight. Yield: 0.075 g (75 %). ^13^C NMR (CP/MAS, δ, ppm): 142.4–125.5 (C=C), 56.8–34.3.

### 2.6. Hydrogenation of Addition PolyDNBD

PolyDNBD (0.2 g, 1.1 mmol monomer units) and *p*-toluenesulfonyl hydrazide (1.4 g, 7.5 mmol) were charged to a two-necked flask equipped with a magnetic stirrer and reflux condenser. The flask was evacuated and filled with argon. Then 10 mL of absolute *o*-xylene were added to the flask, and the mixture was refluxed for 15 h. The precipitate was isolated, soxhleted with chloroform for 3 h, and dried in vacuum at 40–60 °C up to a constant weight. Yield: 0.176 g (88 %). ^13^C NMR (CP/MAS, δ, ppm): 68.4–42.0, 41.6–25.0.

## 3. Results and Discussion

### 3.1. Synthesis of Polymers

**Scheme 1:**
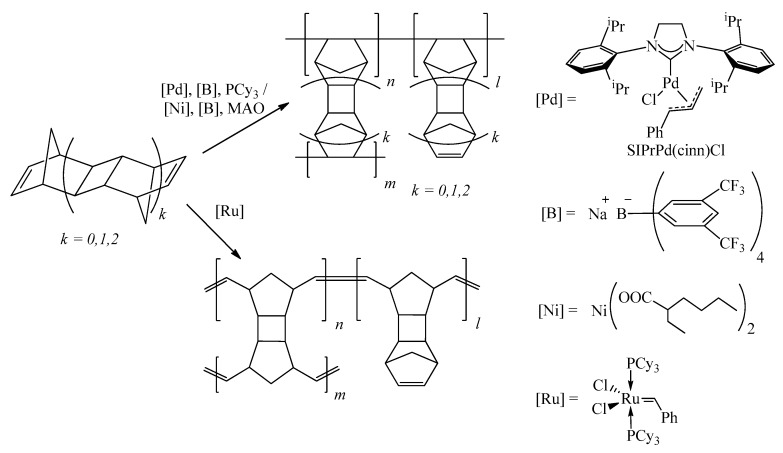
Metathesis and addition polymerization of NBD and its oligomers.

Since norbornadiene-2,5 and its oligomers are bifunctional monomers (they have two polymerizable norbornene double bonds), their polymerization results in the formation of insoluble network polymers. These monomers proved to be highly reactive in metathesis and addition polymerization (Scheme 1). Metathesis polymerization of DNBD was conducted in the presence of the 1st generation Grubbs catalyst leading to a glassy (*T_g_* was higher than the decomposition temperature) and amorphous polymer in a good yield (Table 1). The polymer formed turned out to possess low BET surface area (<10 m^2^/g, see below). Therefore, further investigations of metathesis polymers based on NBD and its oligomers were not performed.

**Figure 2:**
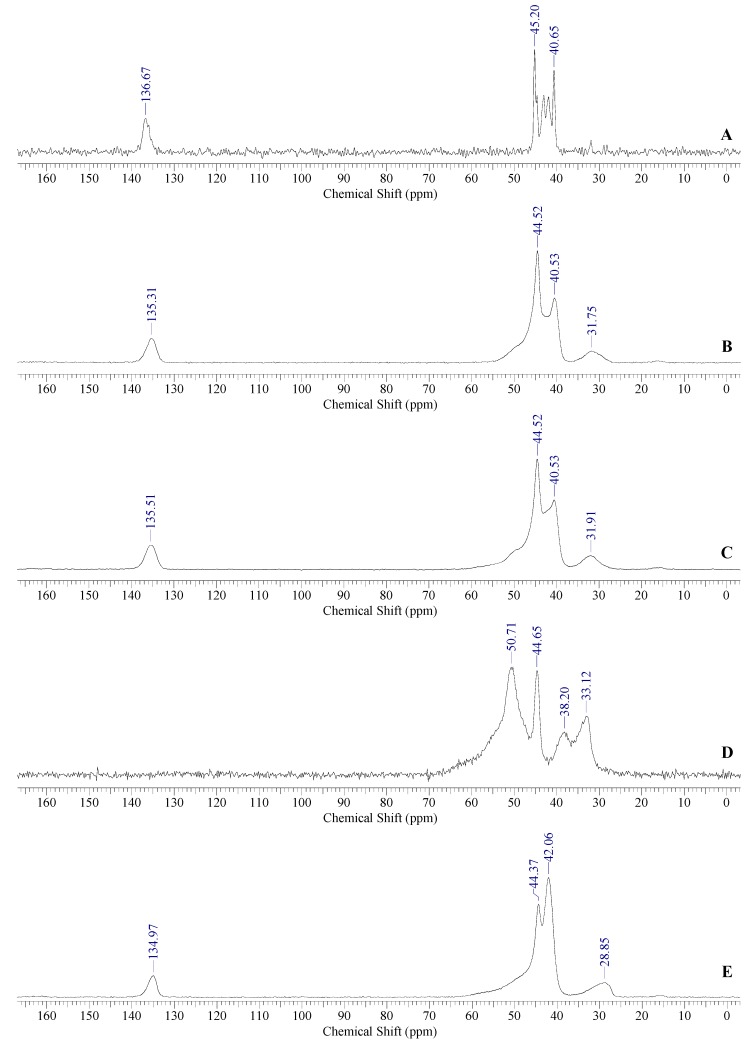
CP/MAS NMR spectra of DNBD (**A**), polyDNBD-es (**B**—synthesized using Ni-catalyst, **C**—synthesized using Pd-catalyst, **D**—hydrogenated polyDNBD synthesized using Pd-catalyst), and polyTNBD synthesized using Pd-catalyst (**E**).

Addition polymerization of DNBD was carried out in the presence of Pd- and Ni-based catalytic systems. As a Pd-catalytic system Pd-*N*-heterocyclic carbene complex (SIPrPd(cinn)Cl, Scheme 1) in combination with NaBARF and tricyclohexylphosphine was applied as one of the most active and stable catalysts in addition polymerization [23,30]. Nickel ethylhexanoate in combination with NaBARF and methylaluminoxane was used as a readily available Ni-based system for addition polymerization. It was found that there is a difference in the process of a polymer formation between polymerizations in the presence of Pd- and Ni-based catalysts. In the case of the former, a few minutes after the initiation a solid precipitate formed, while in the case of the latter a gel formed. Nevertheless, NMR spectra of addition polymers prepared from DNBD in the presence of different catalysts are similar (Figure 2) indicating a close microstructure. Addition homopolymers based on NBD and TNBD were synthesized also using the similar Pd-based catalyst. NBD was as reactive as DNBD, but its polymerization gave some soluble oligomeric fraction (up to 40 mass. %) separated out at the purification stage along with the formation of the insoluble network polymer. The polymerization of TNBD was complicated by its lower solubility in 1,2-dichloroethane (0.13 g/mL) in comparison with DNBD (1.2 g/mL) and as a result the polymerization was performed in more diluted solutions using toluene as a solvent.

**Scheme 2:**
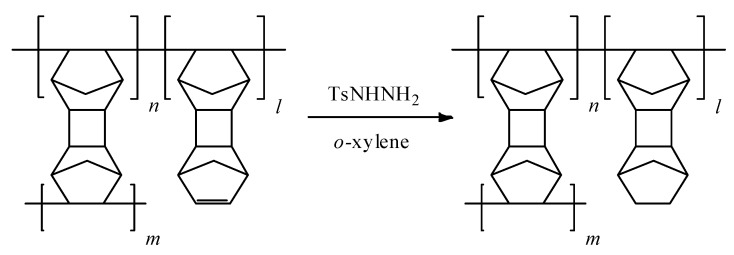
Hydrogenation of addition polyDNBD.

The structure of synthesized homopolymers was examined by CP/MAS NMR (Figure 2) and IR spectroscopy (Figure S1). Despite the fact that addition polymerization results in structures with the saturated nature of main chains, the addition polymers from NBD and its oligomers proved to contain double bonds. This means that not all double bonds were involved in addition polymerization. The amount of double bonds in polymers estimated by ozonation correlates with the monomer size and grows in the row polyNBD < polyDNBD < polyTNBD (Table 1). IR spectra of polymers after ozonation are shown in Figure S2. The amount of double bonds in polyDNBD was estimated by IR spectroscopy and it depended on the nature of catalyst used to obtain the polymer (Table 1). The fully saturated polymers could be readily obtained by the hydrogenation of the polymers formed in addition polymerization. Thus, the hydrogenation of polyDNBD synthesized using Pd-catalyst was done with tosyl hydrazide (Scheme 2). All double bonds were hydrogenated, that was confirmed by ^13^C CP/MAS NMR spectroscopy (Figure 2).

**Figure 3:**
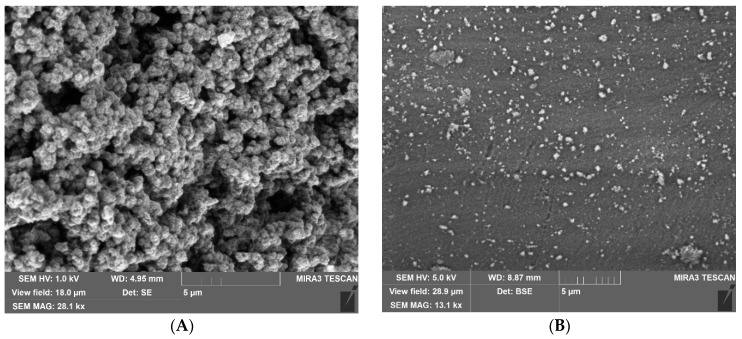
SEM images of polyDNBD-es synthesized over Pd- (**A**) and Ni-catalyst (**B**).

The surface morphology of polyDNBD-es synthesized over Pd- and Ni-catalyst was observed by SEM (Figure 3). Unlike solid polymer synthesized using Ni-catalyst, the polymer synthesized over Pd-catalyst, being a powder, consist of the aggregates of particles with the size of less than 1 μm.

### 3.2. WAXD and TGA Characterization of the Synthesized Polymers

**Figure 4:**
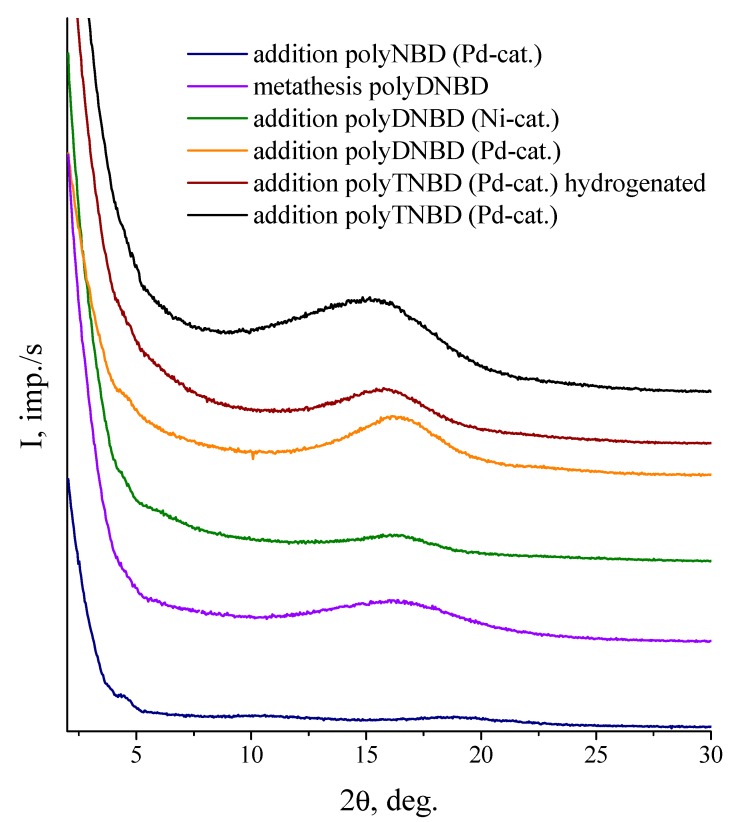
WAXD patterns of homopolymers based on NBD, DNBD and TNBD.

All synthesized addition homopolymers proved to be amorphous (Figure 4). According to WAXD data polyTNBD has the larger *d*-spacing than polyDNBD, which is indicated by a peak shifted at the smaller 2*θ* angle (Table 3). At the same time addition polyDNBD-es obtained over different catalysts differ by *d*-spacing values: the corresponding value for polyDNBD obtained in the presence of Pd-catalyst is higher than that for polyDNBD prepared over Ni-catalyst. This difference in *d*-spacing distances and a more diffused WAXD pattern of addition polyDNBD synthesized with Ni-catalyst clearly indicate different chain packing of polyDNBD polymers obtained over various catalysts. Hydrogenation of polyDNBD led to a polymer with larger *d*-spacing distances in comparison with the d-spacing values for the initial unsaturated polymer and it could be concluded that the hydrogenated polyDNBD has larger free volume and a more porous structure.

**Figure 5:**
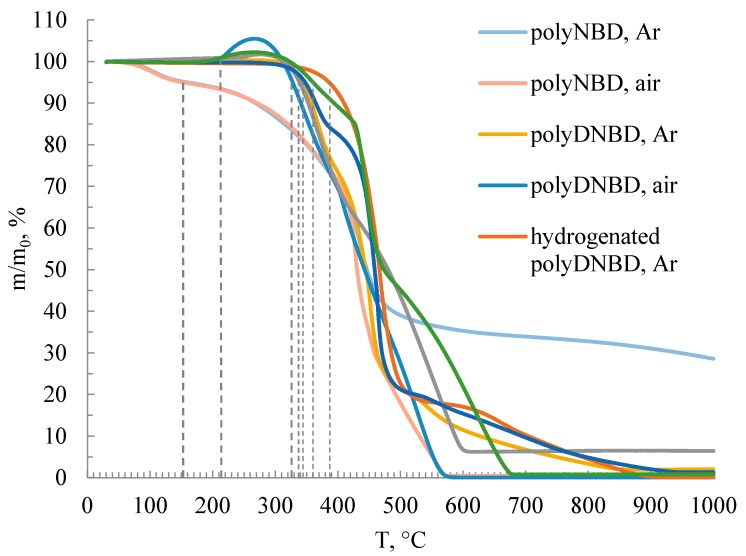
TGA curves of addition polymers from NBD and its oligomers synthesized using Pd-catalyst.

The prepared polymers exhibited high thermal stability under an inert atmosphere (up to 386 °C for 5 % mass loss, Figure 5). In the TGA curve of polyDNBD in air, an increase of the sample mass was revealed at 220–300 °C, which indicated the oxidation of double bonds (see IR spectra in Figure S3). This observation is in contrast to the thermal properties of metathesis polynorbornenes containing internal double bonds in main chains: for them the increase of mass sample was not found in TGA curves in air. This difference is evidence that double bonds in the synthesized polymers are available and they can be readily modified leading to new polymers.

### 3.3. Positron Annihilation Lifetime Spectroscopy (PALS) Study

Interesting results concerning free volume distribution in a polymer were obtained from the analysis of the prepared addition polymers by positron annihilation lifetime spectroscopy (PALS). It was found that in the synthesized polyDNBD, the intensity of smaller free volume elements (*I*_3_) is significantly higher than larger ones (Table 2), in contrast to earlier studied microporous Si-containing addition polynorbornenes [21,22]. At the same time, the intensity of larger free volume elements is very low. The size of free volume elements (*R*_3_ and *R*_4_) is comparable with earlier studied related microporous polynorbornenes [9]. These data are evidence that the prepared addition polymers from NBD and its oligomers possess narrower pore size distribution.

### 3.4. N_2_ and CO_2_ Adsorption Studies

**Figure 6:**
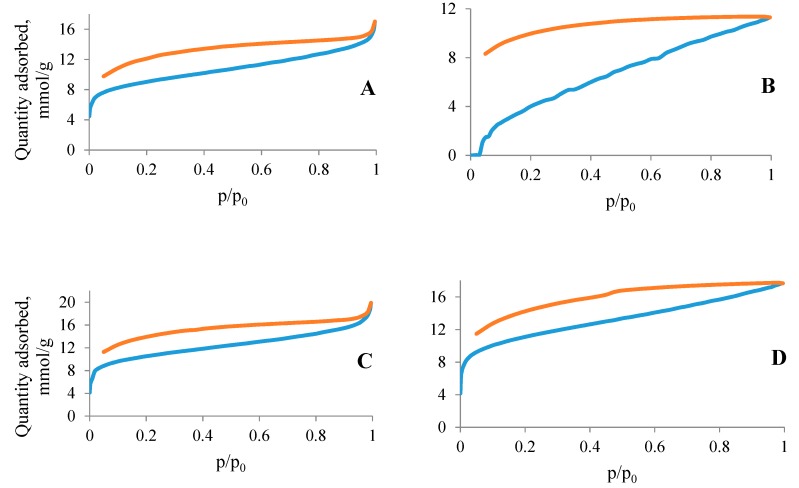
Nitrogen adorption-desorption isotherms of polymers based on norbornadiene and its oligomers: (**A**) polyDNBD (Pd-catalyst); (**B**) polyDNBD (Ni-catalyst); (**C**) hydrogenated polyDNBD (Pd-catalyst); (**D**) polyTNBD (Pd-catalyst).

The characteristics of the porous structure of synthesized polymers was evaluated by low temperature nitrogen and CO_2_ adsorption. Figure 6 and Figure S4 show some adsorption-desorption isotherms of nitrogen for addition polymers from NBD and its oligomers. The shape of isotherms for these polymers is similar to type IV, while the hysteresis loop belongs to H3 type. In the case of all addition polymers prepared herein over Pd-catalyst, there is a high uptake of nitrogen even at low relative pressure. This indicates the presence of micropores for these polymers. At the same time, no microporosity was found for polyDNBD obtained using Ni-catalyst. This polymer was a mesoporous material. Thus, the nature of a catalyst determines the free volume architecture in the considered polymers.

The synthesized addition polymers possess high BET surface area (up to 420–970 m^2^/g, Table 3). The value of BET surface area for the obtained addition polymers depends on the nature of a monomer and the conditions of polymerization. Thus, the highest value was found for the addition polymer from TNBD. This monomer provides the longest interchain linkages within the cross-linked addition polymers based on NBD, its dimer or trimer. It should be noted that, to the best of our knowledge, the value of BET surface area reached in the case of addition polymer from trimer of NBD is the highest one among the values for any polynorbornenes reported so far [16]. The values obtained for specific surface areas are also much higher than a recently reported value for another addition polynorbornene from dicyclopentadiene (*S*_BET_ = 155 m^2^/g) [10]. This difference can be explained by the presence of a bulkier bicyclic group in the monomer and the formation of cross-links during the polymerization, which stabilize the porous architecture of the formed polymers. BET surface area values for the synthesized polymers are close or higher than corresponding values obtained for some other microporous polymers (like PIM-1, PTMSP and TR-polymers) displaying promising properties for membrane gas separation (Table 3). The decrease of the distance between two double bonds in a bifunctional norbornene led to lower BET surface area of the formed addition homopolymers based on these monomers. For example, addition polyDNBD displayed *S*_BET_ as high as 730 m^2^/g. Hydrogenation of the obtained polymers resulted in a noticeable increase of surface area. The synthesis of polymers from NBD and its oligomers in more diluted solutions led to the materials with a little higher BET surface area. This is probably explained by higher conversions of both double bonds of bifunctional NBD oligomers in more diluted solutions. The nature of a catalyst used for a polymer synthesis exerts a stronger influence on porosity of the formed polymers. For instance, for polyDNBD prepared with use of Ni-catalyst, *S*_BET_ was about 400 m^2^/g, while for the same polymer obtained over Pd-catalyst it was more than 700 m^2^/g (Table 3). Furthermore, the nature of a catalyst also governs the pore size distribution. The polyDNBD prepared on Ni-catalyst is a mesoporous material with the dominant pore size of 3.8 nm, while microporosity for this polymer was not found according to t-plot. At the same time, all polymers from NBD or its oligomers prepared in the presence of Pd-catalyst are microporous materials. The true micropore volume and micropore area were estimated by t-plot and the obtained results are presented in Table 3. Based on these data it could be concluded that addition polymers from NBD and its oligomers prepared over Pd-catalyst also have the developed external surface area (Table 3). It is interesting that metathesis polyDNBD, which is an isomer of addition polyDNBD, exhibited extremely low value of BET surface area. This difference in the properties of two isomeric polymers might be explained by various rigidity of polymer main chains. Thus, it was shown that addition polynorbornenes possess more rigid main chains than the similar metathesis polymers [31,32]. So, in the case of addition polymers, a looser packing of polymer chains should be formed due to more rigid main chains, which could not be packed densely.

**Figure 7:**
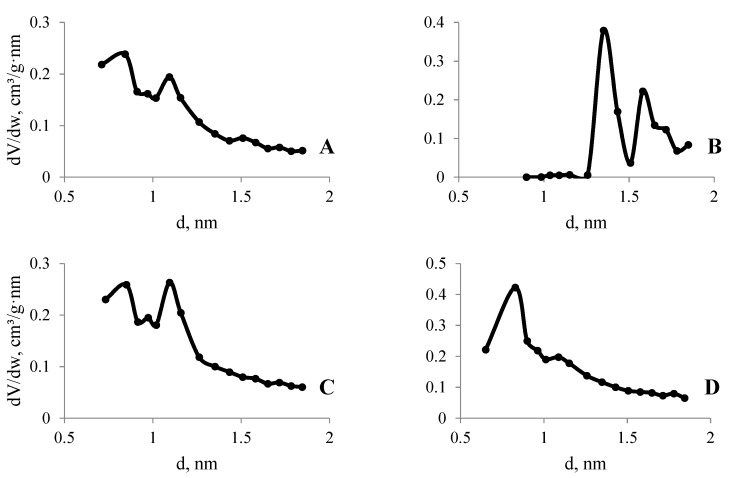
Pore size distribution of polymers based on norbornadiene and its oligomers obtained from N_2_ adsorption data: (**A**) polyDNBD (Pd-catalyst); (**B**) polyDNBD (Ni-catalyst); (**C**) hydrogenated polyDNBD (Pd-catalyst); (**D**) polyTNBD (Pd-catalyst).

Micropore size distribution was calculated using Horvath-Kawazoe method. For these estimations, the model of slit-shape pores was used. It is interesting that for polyDNBD prepared using Pd-catalyst the bimodal micropore size distribution is observed (Figure 7). The maxima are at 0.8 nm (main) and 1.1 nm. After hydrogenation, the intensity of the maximum at 1.1 nm gets higher. At the same time, the micropore size distribution for polyTNBD is more narrow and unimodal, with a mean size of 0.8 nm (Figure 7D). Therefore, it is possible to tune the average micropore size by the choice of the nature of norbornene type monomer. The unimodal and narrow size distribution obtained for polyTNBD can be promising for membrane gas separation or its application in gas storage and polyTNBD may be used as a filler for the composite membranes.

**Figure 8:**
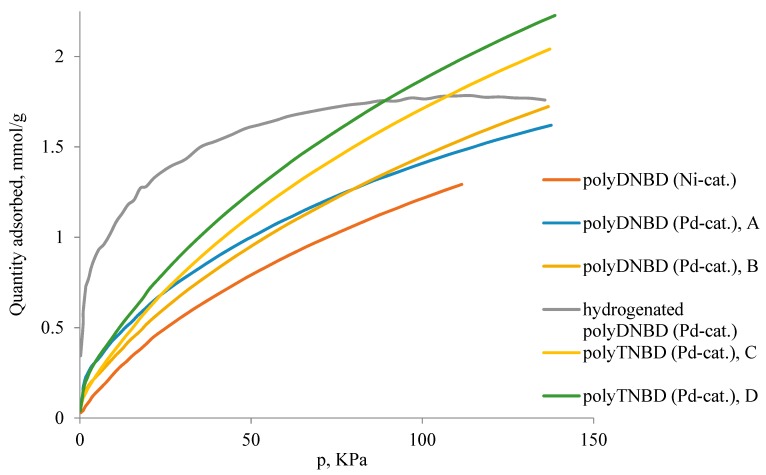
CO_2_ adsorption isotherms (obtained at 0 °C) of polymers based on norbornadiene and its oligomers. A, B, and C, D are the pairs of the similar polymers obtained under different conditions: the concentration of monomer in the reaction mixture was 0.38 M in the case of A, 0.09 M in the case of B, 0.04 M in the case of C and 0.20 M in the case of D.

**Figure 9:**
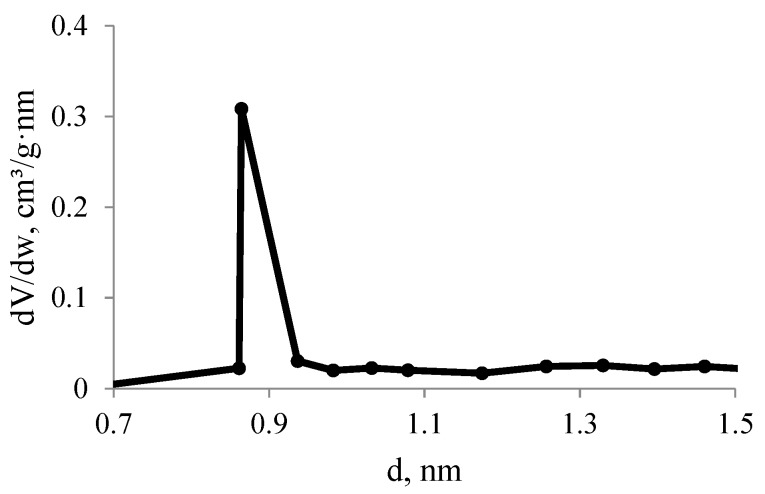
Pore size distribution of polyDNBD (Pd-catalyst) obtained from CO_2_ adsorption data.

The study of the porous structure of the prepared polymers by CO_2_ adsorption revealed that these polymers possess surface areas in the range of 200–403 m^2^/g (Table 4). The shape of adsorption curves is I-type, indicating monomolecular adsorption (Figure 8). In the case of polymers prepared using the Pd-catalyst, a significant adsorption was observed at low CO_2_ pressure (at the beginning of the curves), while for polyDNBD obtained using a Ni-catalytic system, this was not found. The evaluation of the micropore size distribution showed the presence of a very narrow unimodal distribution with the mean pore size of 0.8 nm (Figure 9).

The synthesized polymers gave good CO_2_ uptake at 273 K, which varies from 1.2 to 1.9 mmol/g (Table 4). The highest value of CO_2_ uptake was achieved for polyTNBD, exhibiting also the highest BET surface area from N_2_ adsorption.

## 4. Conclusions

Addition and metathesis homopolymerization of NBD and its oligomers were studied as a way to generate new porous polymer materials. It was shown for the first time that metathesis polymerization of these monomers led to polymers with a low BET surface area, while addition polymerization gave porous materials possessing large BET surface area (up to 970 m^2^/g). The porosity of the addition polymers formed based on NBD and its oligomers can be tuned by the catalyst choice, the nature of monomer and the post-polymerization modification. Therefore, it was found that the Ni-catalytic system gave mesoporous polymers, while addition polymers prepared on the Pd-catalyst were microporous. Specific surface area of polymers increased when the size of the bifunctional monomer became larger. The largest BET surface area as high as 970 m^2^/g was reached in the case of addition polymer from the trimer of norbornadiene-2,5 and this value is the largest among BET surface area values reported for polynorbornenes so far.

## Figures and Tables

**Table 1:** Conditions of polymer synthesis and polymer properties.

Monomer	Synthesis Conditions			Yield, %	Double Bonds Content
	Catalyst ^1^	C, M ^2^	Solvent		
Metathesis polymerization					
DNBD	Ru	0.26	1,2-dichloroethane	100	0.34 ^3^
Addition polymerization					
NBD	Pd	0.20	1,2-dichloroethane	53	0.067 ^4^
DNBD	Pd	0.09	1,2-dichloroethane	100	0.21 ^4^
DNBD	Pd	0.35	1,2-dichloroethane	100	-
DNBD	Pd	0.38	1,2-dichloroethane	100	0.60 ^3^
DNBD	Ni	1.4	toluene	100	0.46 ^3^
TNBD	Pd	0.20	1,2-dichloroethane + toluene (22 vol.%)	96	0.91 ^4^
TNBD	Pd	0.04	1,2-dichloroethane + toluene (4.4 vol.%)	84	-

^1^ Ru means the 1st generation Grubbs catalyst. Pd means SIPrPd(cinn)Cl in combination with NaBARF and PCy_3_. Ni means nickel ethylhexanoate in combinaion with NaBARF, MAO and PCy_3_. ^2^ Monomer concentration in the reaction mixture. ^3^ The amount of norbornene double bonds in the polymer per monomer unit estimated by IR spectroscopy. ^4^ The amount of double bonds per monomer unit estimated by ozonation method.

**Table 2:** Free volume parameters of addition polyDNBD (Pd-cat.) according to positron annihilation lifetime spectroscopy (PALS) in comparison with that of other addition polynorbornenes.

Polymer		*τ*_3_, ns	*I*_3_, %	*τ*_4_, ns	*I*_4_, %	*R*_3_, Å	*R*_4_, Å
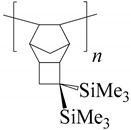	[21]	3.8	11	11.7	36	4.2	7.3
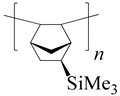	[22]	2.4	12	6.3	36	3.3	5.4
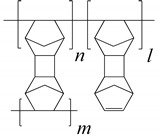 (Pd-cat.)		1.7	35	8.0	4.8	2.5	6.2

**Table 3:** N_2_ adsorption characteristics of polymers based on norbornadiene and its oligomers.

Polymer	*C*, M ^1^	*S*_BET_, m^2^/g	Total Pore Volume, cm^3^/g	t-Plot Micropore Area, m^2^/g	t-Plot External Surface Area, m^2^/g	t-Plot Micropore Volume, cm^3^/g	BJH-Adsorption Mesopore Volume, cm^3^/g	HK-Model ^2^		DR-Model ^3^	
								*V*_max_, cm^3^/g	*d*, nm	*S*, m^2^/g	*V*_mon_, cm^3^/g
addition polyNBD (Pd-cat.)	0.20	<10	-	-	-	-	-	-	-	-	-
metathesis polyDNBD	0.26	<10	-	-	-	-	-	-	-	-	-
addition polyDNBD (Ni-cat.)	1.4	420	0.39	-	631	-	0.27	0.097	1.4	1245	286
addition polyDNBD (Pd-cat.)	0.09	827	0.59	362	465	0.15	0.24	0.34	0.77	942	216
	0.35	736	0.54	355	381	0.14	0.24	0.29	0.73	820	288
	0.38	730	0.59	348	383	0.15	0.28	0.29	0.71	824	189
hydrogenated addition polyDNBD (Pd-cat.)	0.38	814	0.72	378	437	0.16	0.35	0.32	0.73	953	219
addition polyTNBD (Pd-cat.)	0.20	969	0.79	430	538	0.18	0.39	0.38	0.72	1072	246
PIM-1 [33,34]	-	850–860	0.78	-	-	-	-	-	-	-	-
TR-1-450 [7]	-	510	-	-	-	-	-	-	-	-	-
PTMSP [35]	-	780	-	-	-	-	-	-	-	-	-

^1^ The concentration of monomer in the reaction mixture while the polymerization. ^2^ Horvath-Kawazoe model. *V*_max_ means maximum pore volume, *d* means median pore width. ^3^ Dubinin-Radushkevich model. *S* means micropore surface area, *V*_mon_ means monolayer capacity.

**Table 4:** CO_2_ adsorption characteristics of polymers based on norbornadiene and its oligomers.

Polymer	*C*^1^, M	*S*_L_^2^, m^2^/g	CO_2_ Uptake, mmol/g
addition polyDNBD (Pd-cat.)	0.09	314	1.46
	0.38	254	1.42
addition polyDNBD (Ni-cat.)	1.4	276	1.22
hydrogenated addition polyDNBD (Pd-cat.)	0.38	202	1.77
addition polyTNBD (Pd-cat.)	0.20	403	1.89

^1^ The concentration of monomer in the reaction mixture while the polymerization. ^2^ Surface area calculated by Langmuir equation.

## Supplementary Materials

The following (Figures S1–S4) are available online at http://www.mdpi.com/2073-4360/10/12/1382/s1, Figure S1: IR spectra of homopolymers based on NBD and its oligomers, Figure S2: IR spectra of ozonated homopolymers based on NBD and its oligomers, Figure S3: IR spectra of oxidized and initial polyDNBD (Pd-catalyst). The oxidation was performed by heating of the sample in air for 30 min, Figure S4: Nitrogen adsorption-desorption isotherms of homopolymers from NBD and its oligomers.

## References

[1] Morris R.E., Wheatley P.S. (2008). Gas Storage in Nanoporous Materials. Angew. Chem. Int. Ed..

[2] Low Z.-X., Budd P.M., McKeown N.B., Patterson D.A. (2018). Gas Permeation Properties, Physical Aging, and Its Mitigation in High Free Volume Glassy Polymers. Chem. Rev..

[3] Zhou H.-C., Long J.R., Yaghi O.M. (2012). Introduction to Metal–Organic Frameworks. Chem. Rev..

[4] Furukawa H., Yaghi O.M. (2009). Storage of Hydrogen, Methane, and Carbon Dioxide in Highly Porous Covalent Organic Frameworks for Clean Energy Applications. J. Am. Chem. Soc..

[5] Takada K., Matsuya H., Masuda T., Higashimura T. (1985). Gas permeability of polyacetylenes carrying substituents. J. Appl. Polym. Sci..

[6] McKeown N.B., Budd P.M. (2006). Polymers of intrinsic microporosity (PIMs): Organic materials for membrane separations, heterogeneous catalysis and hydrogen storage. Chem. Soc. Rev..

[7] Park H.B., Jung C.H., Lee Y.M., Hill A.J., Pas S.J., Mudie S.T., Wagner E.V., Freeman B.D., Cookson D.J. (2007). Polymers with Cavities Tuned for Fast Selective Transport of Small Molecules and Ions. Science.

[8] Li G., Liu Q., Xia B., Huang J., Li S., Guan Y., Zhou H., Liao B., Zhou Z., Liu B. (2017). Synthesis of stable metal-containing porous organic polymers for gas storage. Eur. Polym. J..

[9] Chapala P.P., Bermeshev M.V., Gavrilova N.N. (2017). Microporous structure of highly permeable additive silicon-containing polytricyclononenes. Polym. Sci. Ser. A.

[10] Wozniak A.I., Bermesheva E.V., Gavrilova N.N., Ilyasov I.R., Nechaev M.S., Asachenko A.F., Topchiy M.A., Gribanov P.S., Bermeshev M.V. (2018). Addition Homo- and Copolymerizations of Dicyclopentadiene and 5-n-Hexylnorbornene in the Presence of Pd-N-Heterocyclic Carbene Complexes. Macromol. Chem. Phys..

[11] Du N., Dal-Cin M.M., Robertson G.P., Guiver M.D. (2012). Decarboxylation-Induced Cross-Linking of Polymers of Intrinsic Microporosity (PIMs) for Membrane Gas Separation. Macromolecules.

[12] Liu Q., Li G., Tang Z., Chen L., Liao B., Ou B., Zhou Z., Zhou H. (2017). Design and synthesis of conjugated polymers of tunable pore size distribution. Mater. Chem. Phys..

[13] Lee J.-Y., Wood C.D., Bradshaw D., Rosseinsky M.J., Cooper A.I. (2006). Hydrogen adsorption in microporous hypercrosslinked polymers. Chem. Commun..

[14] Ivin K.J., Mol J.C., Mol K.J.I.C. (1997). 11—Ring-Opening Metathesis Polymerization: General Aspects. Olefin Metathesis and Metathesis Polymerization (2).

[15] Blank F., Janiak C. (2009). Metal catalysts for the vinyl/addition polymerization of norbornene. Coord. Chem. Rev..

[16] Bermeshev M.V., Chapala P.P. (2018). Addition polymerization of functionalized norbornenes as a powerful tool for assembling molecular moieties of new polymers with versatile properties. Prog. Polym. Sci..

[17] Gaylord N.G., Deshpande A.B., Mandal B.M., Martan M. (1977). Poly-2,3- and 2,7-Bicyclo[2.2.1]hept-2-enes: Preparation and Structures of Polynorbornenes. J. Macromol. Sci. A.

[18] Bermeshev M.V., Bulgakov B.A., Genaev A.M., Kostina J.V., Bondarenko G.N., Finkelshtein E.S. (2014). Cationic Polymerization of Norbornene Derivatives in the Presence of Boranes. Macromolecules.

[19] Finkelshtein E., Gringolts M., Bermeshev M., Chapala P., Rogan Y., Yampolskii Y., Finkelshtein E. (2017). Polynorbornenes. Membrane Materials for Gas and Vapor Separation.

[20] Finkelshtein E.S., Bermeshev M.V., Gringolts M.L., Starannikova L.E., Yampolskii Y.P. (2011). Substituted polynorbornenes as promising materials for gas separation membranes. Russ. Chem. Rev..

[21] Chapala P.P., Bermeshev M.V., Starannikova L.E., Belov N.A., Ryzhikh V.E., Shantarovich V.P., Lakhtin V.G., Gavrilova N.N., Yampolskii Y.P., Finkelshtein E.S. (2015). A Novel, Highly Gas-Permeable Polymer Representing a New Class of Silicon-Containing Polynorbornens As Efficient Membrane Materials. Macromolecules.

[22] Alentiev D.A., Bermeshev M.V., Starannikova L.E., Bermesheva E.V., Shantarovich V.P., Bekeshev V.G., Yampolskii Y.P., Finkelshtein E.S. (2018). Stereoselective synthesis and polymerization of Exo-5-trimethylsilylnorbornene. J. Polym. Sci. Part A Polym. Chem..

[23] Alentiev D.A., Egorova E.S., Bermeshev M.V., Starannikova L.E., Topchiy M.A., Asachenko A.F., Gribanov P.S., Nechaev M.S., Yampolskii Y.P., Finkelshtein E.S. (2018). Janus tricyclononene polymers bearing tri(n-alkoxy)silyl side groups for membrane gas separation. J. Mater. Chem. A.

[24] Viciu M.S., Navarro O., Germaneau R.F., Kelly R.A., Sommer W., Marion N., Stevens E.D., Cavallo L., Nolan S.P. (2004). Synthetic and Structural Studies of (NHC)Pd(allyl)Cl Complexes (NHC = *N*-heterocyclic carbene). Organometallics.

[25] Marion N., Navarro O., Mei J., Stevens E.D., Scott N.M., Nolan S.P. (2006). Modified (NHC)Pd(allyl)Cl (NHC = *N*-Heterocyclic Carbene) Complexes for Room-Temperature Suzuki−Miyaura and Buchwald−Hartwig Reactions. J. Am. Chem. Soc..

[26] Dzhemilev U.M., Popod’ko N.R., Kozlova E.V. (1999). Metallokomleksny kataliz v organicheskom sinteze. Alitsiklicheskiye soedineniya.

[27] Lisitsin D.M., Poznyak T.I., Razumovskii S.D. (1976). Mathematical modeling of the reaction of ozone with some organic compounds in a continuous operation bubbling reactor. Kinet. Katalis.

[28] Tao S.J. (1972). Positronium Annihilation in Molecular Substances. J. Chem. Phys..

[29] Eldrup M., Lightbody D., Sherwood J.N. (1981). The temperature dependence of positron lifetimes in solid pivalic acid. Chem. Phys..

[30] Alentiev D.A., Korchagina S.A., Finkelshtein E.S., Nechaev M.S., Asachenko A.F., Topchiy M.A., Gribanov P.S., Bermeshev M.V. (2018). Addition homo- and copolymerization of 3-triethoxysilyltricyclo[4.2.1.0^2,5^]non-7-ene. Russ. Chem. Bull..

[31] Yevlampieva N., Bermeshev M., Vezo O., Chapala P., Il’yasova Y. (2018). Metathesis and additive poly(tricyclononenes) with geminal trimethylsilyl side groups: Chain rigidity, molecular and thin film properties. J. Polym. Res..

[32] Yevlampieva N.P., Bermeshev M.V., Komolkin A.V., Vezo O.S., Chapala P.P., Il’yasova Y.V. (2017). The equilibrium and kinetic rigidity of additive poly(trimethylsilyltricyclononenes) with one and two Si(CH3)3 groups in monomer unit. Polym. Sci. Ser. A.

[33] Budd P.M., Ghanem B.S., Makhseed S., McKeown N.B., Msayib K.J., Tattershall C.E. (2004). Polymers of intrinsic microporosity (PIMs): Robust, solution-processable, organic nanoporous materials. Chem. Commun..

[34] Budd P.M., Elabas E.S., Ghanem B.S., Makhseed S., McKeown N.B., Msayib K.J., Tattershall C.E., Wang D. (2004). Solution-Processed, Organophilic Membrane Derived from a Polymer of Intrinsic Microporosity. Adv. Mater..

[35] Thomas S., Pinnau I., Du N., Guiver M.D. (2009). Pure- and mixed-gas permeation properties of a microporous spirobisindane-based ladder polymer (PIM-1). J. Membr. Sci..

