# Supplementary material for: Microporous Materials Based on Norbornadiene-Based Cross-Linked Polymers
Source: Polymers (Basel). 2018 Dec 13;10(12):1382. doi: 10.3390/polym10121382 (PMC6401845; doi:10.3390/polym10121382)
Supplement: Supplementary file 1 [file polymers-10-01382-s001.pdf]

Microporous materials based on addition homopolymers from norbornadiene, its dimer and trimer

Dmitry A. Alentiev[1], Dariya M. Dzhaparidze[1,2], Natalia N. Gavrilova[3], Victor P. Shantarovich[4], Elena V. Kiseleva[4], Maxim A. Topchiy[1], Andrey F. Asachenko[1], Pavel S. Gribanov[1], Mikhail S. Nechaev[1,5], Maxim V. Bermeshev[1,3]

[1] A.V. Topchiev Institute of Petrochemical Synthesis, Russian Academy of Sciences, 29 Leninsky prospekt, 119991 Moscow, Russia

[2] A.N. Kosygin Russian State University, 33-1 Sadovnicheskaya st., 117997 Moscow, Russia

[3] D.I. Mendeleev University of Chemical Technology of Russia, 9 Miusskaya sq., 125047 Moscow, Russia

[4] N.N. Semenov Institute of Chemical Physics, Russian Academy of Sciences, 4 Kosygina st., 119991 Moscow, Russia

[5] M.V. Lomonosov Moscow State University, Chemistry Department, 1-3 Leninskie gory, 119991 Moscow, Russia

---

1. Supplementary figures

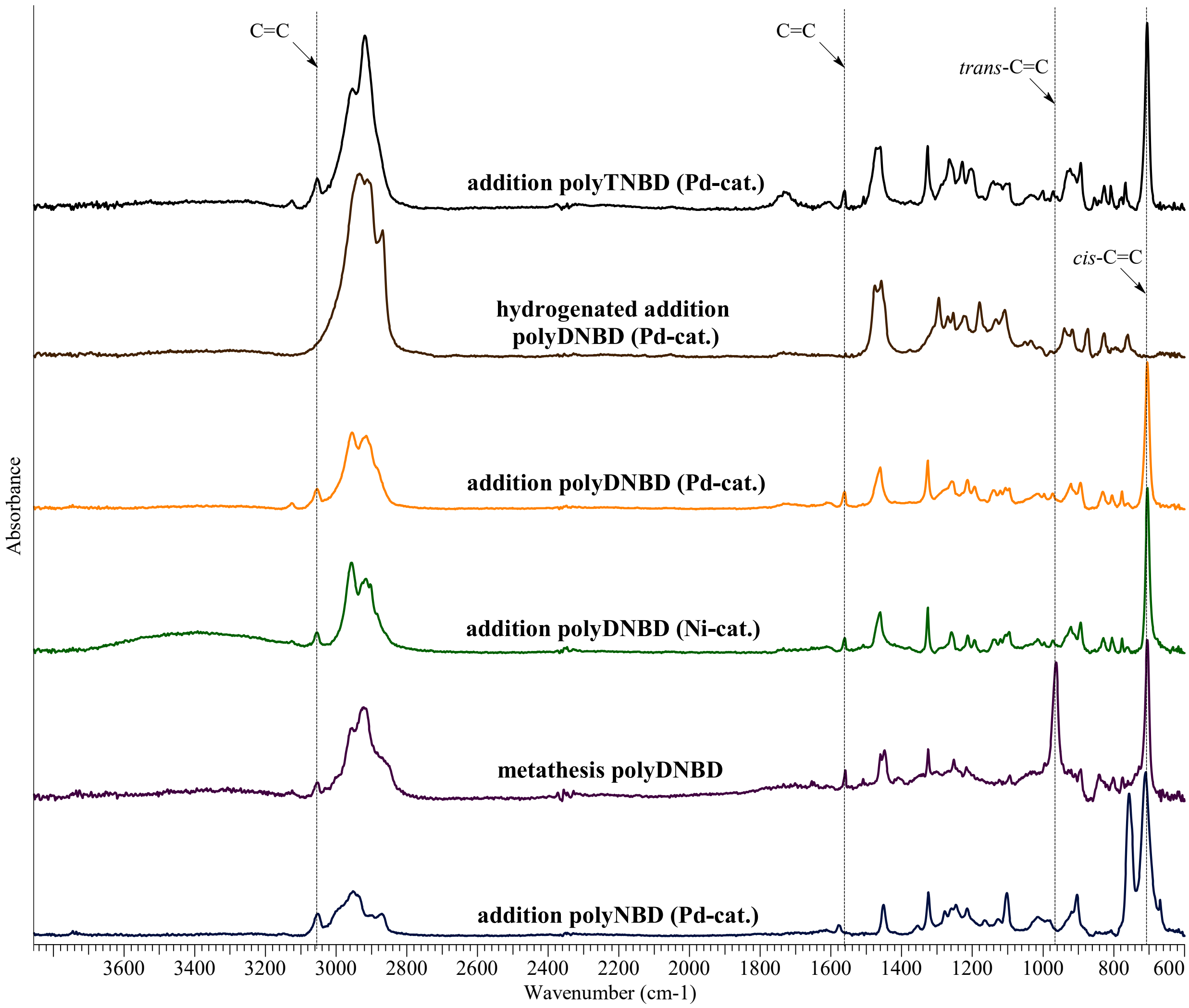

Figure S1. IR spectra of homopolymers based on NBD and its oligomers.

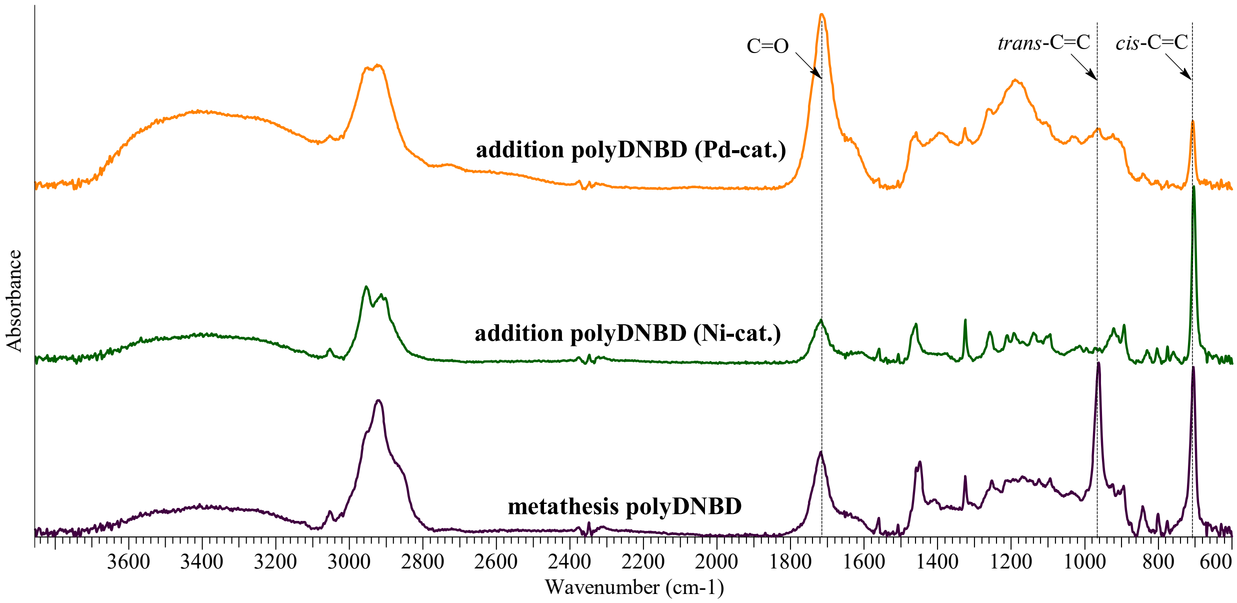

Figure S2. IR spectra of ozonated homopolymers based on NBD and its oligomers.

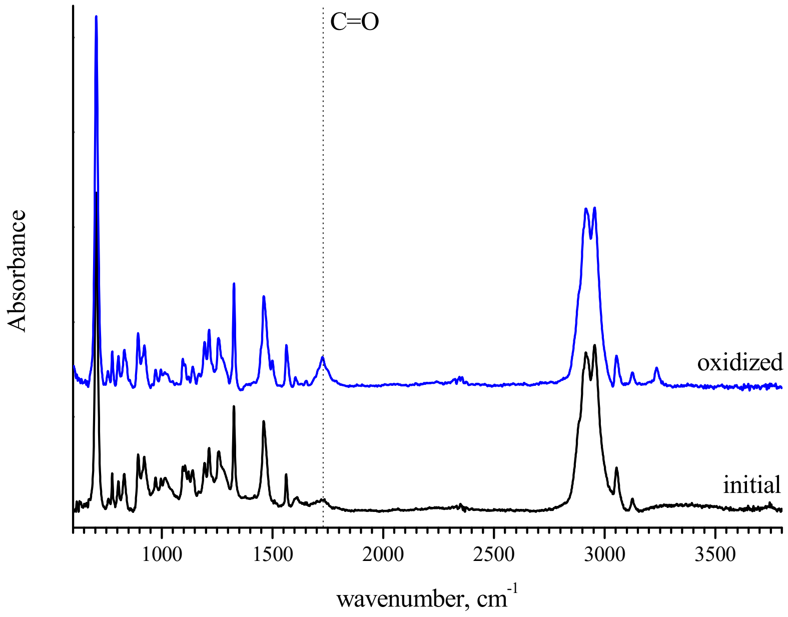

Figure S3. IR spectra of oxidized and initial polyDNBD (Pd-catalyst). The oxidation was performed by heating of the sample in air for 30 min.

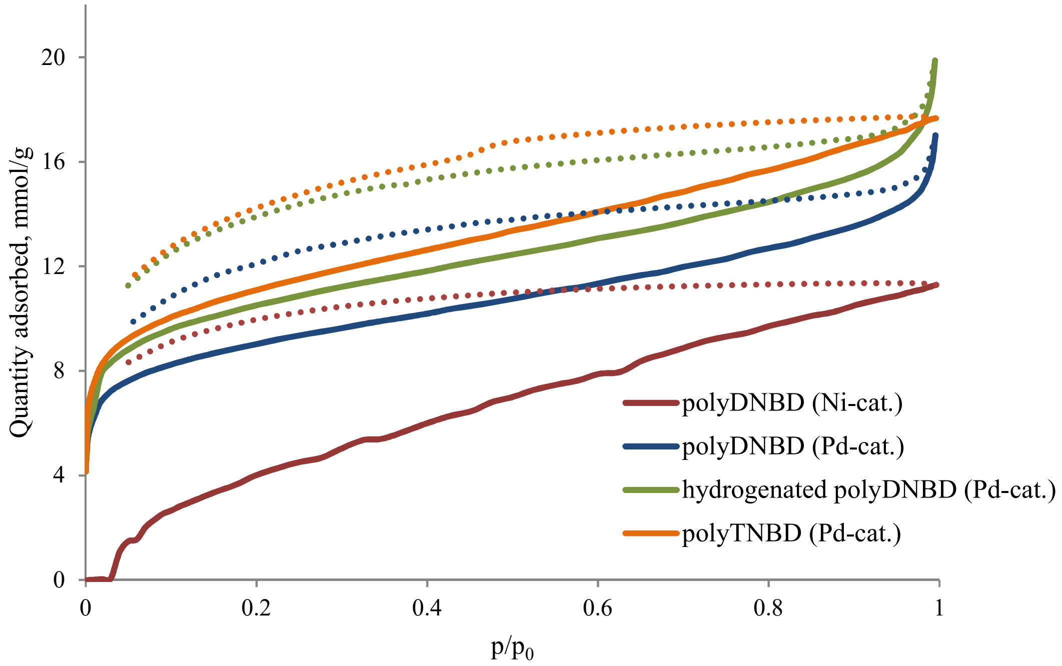

Figure S4. Nitrogen adsorption-desorption isotherms of homopolymers from NBD and its oligomers.
